# Dietary Supplementation of Zinc Oxide Quantum Dots Protective Against *Clostridium perfringens* Induced Negative Effects in Broilers

**DOI:** 10.3390/toxins17060272

**Published:** 2025-05-29

**Authors:** Lei Shi, Qin-Jian Niu, Hao-Hua Xu, Yu-Xuan Huang, Yu-Wei Zhao, Alainaa Refaie, Lv-Hui Sun, Zhang-Chao Deng

**Affiliations:** 1 State Key Laboratory of Agricultural Microbiology, Hubei Hongshan Laboratory, Frontiers Science Center for Animal Breeding and Sustainable Production, Key Laboratory of Smart Farming Technology for Agricultural Animals of Ministry of Agriculture and Rural Affairs, College of Animal Science and Technology, Huazhong Agricultural University, Wuhan 430070, China; shilei123@webmail.hzau.edu.cn (L.S.); niuqinjian@webmail.hzau.edu.cn (Q.-J.N.); haohuaxu@webmail.hzau.edu.cn (H.-H.X.); huangyuxuan@webmail.hzau.edu.cn (Y.-X.H.); alainaa.refaie@webmail.hzau.edu.cn (A.R.); 2 Zhongke Jichuang New Material Technology Co., Ltd., Chengdu 610000, China; 3 Yantai Longda Breeding Co., Ltd., Yantai 265209, China; zhaoyuwei@longdameishi.com

**Keywords:** *Clostridium perfringens*, zinc oxide quantum dots, intestinal health, antioxidant, gut microbiome

## Abstract

*Clostridium perfringens* is a major cause of necrotizing enteritis in chickens. This study aimed to investigate the effects of zinc oxide quantum dots (ZnO-QDs) on growth performance, redox status, and gut microbiota in broilers challenged with *C. perfringens*. A total of 320 1-day-old chicks were divided into five groups: negative control (NC) without treatment; positive control (PC) infected with *C. perfringens*; and the other three groups (40, 80, and 120 Zn) were given ZnO-QDs at doses of 40, 80, and 120 mg/kg, respectively, under *C. perfringens* infection, respectively. The results show that, compared to the NC group, the PC group exhibited negative effects on growth performance, intestinal morphology, and antioxidant status in broilers. However, compared to the PC group, 120 mg Zn increased (*p* < 0.05) the body weight of broilers at 21 days, while 40 mg Zn reduced (*p* < 0.05) serum diamine oxidase activity. The intestinal macroscopic evaluation showed that the PC group had the highest lesion scores, whereas the 120 mg Zn group exhibited the lowest lesion score. Meanwhile, compared to the PC group, the 40 mg Zn group had higher (*p* < 0.05) CAT and GPX activities and a lower (*p* < 0.05) MDA concentration. Moreover, the 40 mg Zn group up-regulated (*p* < 0.05) the gene expression of *Cathelicidin-1*, *IL-10*, *Claudin-1*, and *MLCK* in the jejunum. Furthermore, the 120 mg Zn group increased (*p* < 0.05) the abundance of *Blautia*, *Parasutterella*, and *Lachnospiraceae FCS020* in the cecum. In conclusion, ZnO-QDs exerted a beneficial effect on improving growth performance and overall health in broilers under *C. perfringens* infection, potentially by regulating redox balance and gut microbiota.

## 1. Introduction

Necrotic enteritis (NE) is a bacterial enteric poultry disease that adversely affects the growth performance of birds and results in significant economic losses in the broiler industry [1]. Acute or clinical NE manifests as sudden flock death, with mortality rates surging to 50%, while the more common subclinical form of NE also poses substantial challenges [2,3]. Statistical data indicate that NE causes approximately USD 6 billion in annual economic loss for the global poultry industry [4]. *Clostridium perfringens* is the primary causative pathogen of NE. The proliferation of this pathogen in the chicken intestine leads to toxin production, which induces necrotic mucosal lesions in the gut and may manifest as either acute or subclinical disease entities [5,6]. Therefore, developing effective strategies to mitigate *C. perfringens* infection is critical for the management and prevention of NE.

Antibiotics were routinely used to control the outbreaks of NE induced by causative pathogens, especially *C. perfringens*, in poultry flocks [7]. However, growing concerns regarding antibiotic resistance have prompted regulatory bans in numerous regions to prohibit antibiotic use, consequently triggering a resurgence of NE outbreaks [8,9]. As the poultry industry increasingly moves away from in-feed antibiotics, alternative strategies are required to address the persistent threat of NE. Zinc oxide (ZnO), a widely used feed additive, not only satisfies zinc nutritional requirements but also demonstrates antimicrobial and growth-promoting effects [10,11]. It is recognized as a “Generally Recognized as Safe” (GRAS) substance by the U.S. Food and Drug Administration (21CFR182.8991). Moreover, ZnO nanoparticles exhibit greater biological activity compared to conventional zinc sources and enhance the performance and antioxidant defense of chickens [12,13]. Previous studies have demonstrated that variations in ZnO particle size and shape result in differing degrees of antibacterial activity [14]. Compared with traditional ZnO (particle size > 1 μm), ZnO nanoparticles (mean particle size 50–70 nm) reduced the growth rate of *Staphylococcus aureus* by 50%, suggesting that antibacterial efficacy improves as the particle size decreases [15,16]. This phenomenon may be attributed to the small size and high surface-to-volume ratio of nanoparticles, which enhance their interaction with bacterial cells [17].

Zinc oxide quantum dots (ZnO-QDs), as novel fluorescent nanofunctional materials with a particle size under 10 nm, exhibit remarkable antimicrobial performance. They are easy to prepare, cost-effective, readily available, and nontoxic [18,19]. Our previous study indicated that a diet supplemented with 80 mg Zn/kg ZnO-QDs improved the broilers’ growth and sustained the normal intestinal structure and function [20]. Additionally, it has been reported that ZnO-QDs provide protective effects against disease in poultry by inhibiting pathogenic bacterial growth [19,21]. However, the antibacterial activity of ZnO-QDs against *C. perfringens* infection in broilers remains unclear. Therefore, this study aimed to evaluate the efficacy of dietary supplementation with ZnO-QDs, comparing with *C. perfringens* infection, on growth performance, redox homeostasis, gut health, and the microbial composition of broilers.

## 2. Results

### 2.1. In Vitro Antibacterial Activities of ZnO-QDs

**Figure 1:**
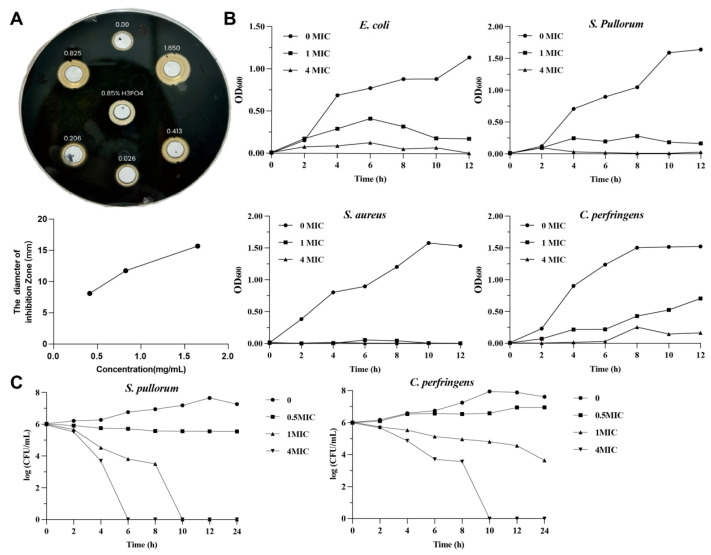
Bacteriostatic effect of ZnO-QDs on *E. coli*, *S. aureus*, *S. Pullorum*, and *C. perfringens* in vitro. The diameter of the inhibition zone of *C. perfringens* (**A**); bacteriostatic effect of 0, 1, and 4 MIC ZnO-QDs (**B**); time-kill curves of 0, 0.5, 1, and 4 MIC ZnO-QDs (**C**). Values are means of one to three independent experiments.

Antibacterial solutions containing ZnO-QDs or ZnO were prepared and mixed with four bacterial suspensions to evaluate their antibacterial activity. The MIC values of the ZnO-QDs for *E. coli*, *S. Pulorum*, *S. aureus*, and *C. perfringens* were 0.413, 0.052, 0.026, and 0.413 mg/mL, respectively. Meanwhile, the MIC values of ZnO for *E. coli*, *S. Pulorum*, *S. aureus*, and *C. perfringens* were 3.800, 0.059, 0.238, and 1.900 mg/mL (Table 1). These results indicate that ZnO-QDs exhibit higher antibacterial activity than traditional ZnO. Further analysis of the inhibition zones for *C. perfringens* showed that the diameters of the inhibition zones increased as the concentration of the ZnO-QDs was raised (Figure 1A). Moreover, bacterial growth monitoring confirmed that the ZnO-QDs effectively suppressed the growth of all four bacterial strains (Figure 1B). Specifically, a 4 MIC dose of ZnO-QDs eradicated *S. pullorum* within 6 h and *C. perfringens* within 10 h, respectively (Figure 1C).

### 2.2. Growth Performance

As shown in Table 2, compared to the NC group, the PC group significantly reduced (*p* < 0.05) body weight at 21 days old, while the 40 and 120 mg Zn groups significantly increased (*p* < 0.05) body weight. Meanwhile, the PC group trended to increase (*p* = 0.071) the FCR during 1 to 28 days relative to the NC group. Notably, the effect was alleviated by the dietary ZnO-QD supplementation. Moreover, no differences were found in the ADG and ADFI among the five groups.

### 2.3. Intestinal Health and Serum Biochemistry

**Figure 2:**
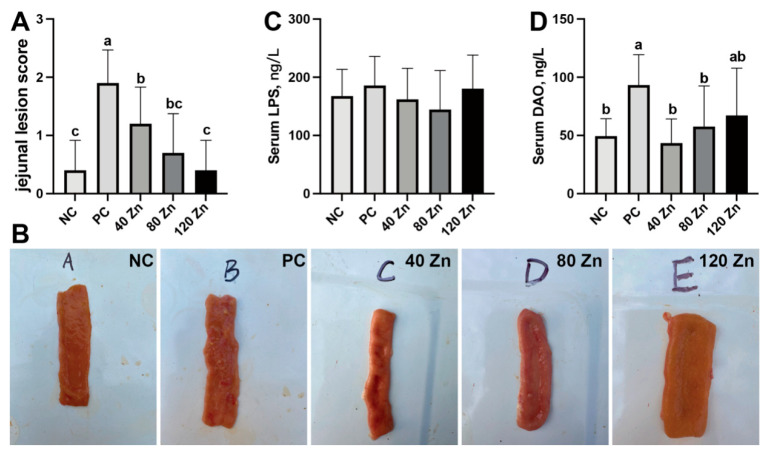
Impact of ZnO-QDs on the lesion score of jejunal and serum LPS and DAO content of broilers infected by *C. perfringens*. The jejunal lesion score (**A**), gut morphology (**B**), LPS and DAO in the serum (**C**,**D**). Values are means ± SD, n = 7–10. Different letters between groups represent significant differences, *p* < 0.05. NC, negative control without treatment; PC, positive control with oral gavage of *C. perfringens*; 40 Zn, 80 Zn, and 120 Zn, dietary supplementation of 40, 80, and 120 mg/kg ZnO-QDs with oral gavage of *C. perfringens*.

Compared to the NC group, the PC group exhibited ulceration, a thinner intestinal wall of the jejunum, and an increased (*p* < 0.05) lesion score (Figure 2A,B). Notably, dietary supplementation of ZnO-QDs significantly alleviated the intestinal lesions, with the 120 mg Zn dosage exhibiting the most pronounced ameliorative effect. Although the serum LPS showed no differences among the groups (Figure 2C), further serum DAO analysis showed that the PC group significantly increased (*p* < 0.05) the DAO contents when compared to the NC group, while the 40 and 80 mg Zn groups significantly mitigated (*p* < 0.05) this elevation (Figure 2D).

### 2.4. Jejunal Redox Status

The results of the indicators of jejunal redox status are shown in Table 3. Compared to the NC group, the PC group decreased (*p* < 0.05) the CAT activity and exhibited a tendency toward higher MDA content; however, the groups with dietary supplementation of 40 or 80 mg Zn alleviated the changes in MDA induced by *C. perfringens* infection. A significant decrease in SOD activity was observed (*p* < 0.05) in the PC group, while it was moderately increased by dietary ZnO-QD supplementation, although this was not statistically significant. Notably, although the PC group did not alter the GPX activity, it was enhanced (*p* < 0.05) by dietary supplementation with 40 and 120 mg Zn/kg ZnO-QDs.

### 2.5. The mRNA Levels of Antimicrobial Peptide-, Cytokine-, and Tight Junction-Related Genes

**Figure 3:**
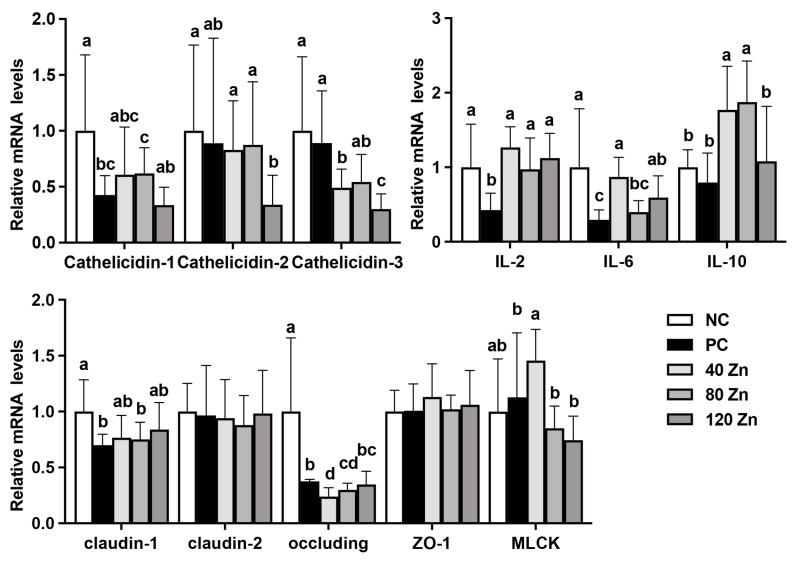
Effect of ZnO-QDs on antimicrobial peptide, cytokine, and tight junction gene relative mRNA levels in the jejunum of broilers infected by *C. perfringens*. Values are means ± SD, n = 7–10. Different letters between groups represent significant differences, *p* < 0.05. IL, interleukin; ZO-1, tight junction protein 1; MLCK, myosin light chain kinase; NC, negative control without treatment; PC, positive control with oral gavage of *C. perfringens*; 40 Zn, 80 Zn, and 120 Zn, dietary supplementation with 40, 80, and 120 mg/kg ZnO-QDs with oral gavage of *C. perfringens*.

As presented in Figure 3, compared with the NC group, the PC group showed down-regulation (*p* < 0.05) of *Cathelicidin-1* expression at the mRNA level, and the 40 and 80 mg Zn groups partially mitigated this decrease. Although no differences were found in the mRNA levels of *Cathelicidin-2* and *Cathelicidin-3* between the NC and PC groups, the 120 mg Zn group significantly reduced (*p* < 0.05) the mRNA expression of *Cathelicidin-1*, *Cathelicidin-2*, and *Cathelicidin-3* genes when compared to the NC or PC groups. Additionally, compared with the NC group, the *IL-2* and *IL-6* mRNA levels were significantly reduced (*p* < 0.05) in the PC group, while the 40 and 120 mg Zn groups significantly elevated (*p* < 0.05) the expression of *IL-2* and *IL-6* genes. Meanwhile, the 40 and 80 mg Zn groups significantly up-regulated (*p* < 0.05) *IL-10* gene expression when compared to the NC or PC groups. Notably, *C. perfringens* infection significantly reduced (*p* < 0.05) the expression of *Claudin-1* and *Occludin* genes, while the 40 and 120 mg Zn groups alleviated the decrease in *Claudin-1* expression. Additionally, the 40 mg Zn group markedly up-regulated (*p* < 0.05) the mRNA expression of *MLCK* when compared to the NC group.

### 2.6. Gut Microbiota

**Figure 4:**
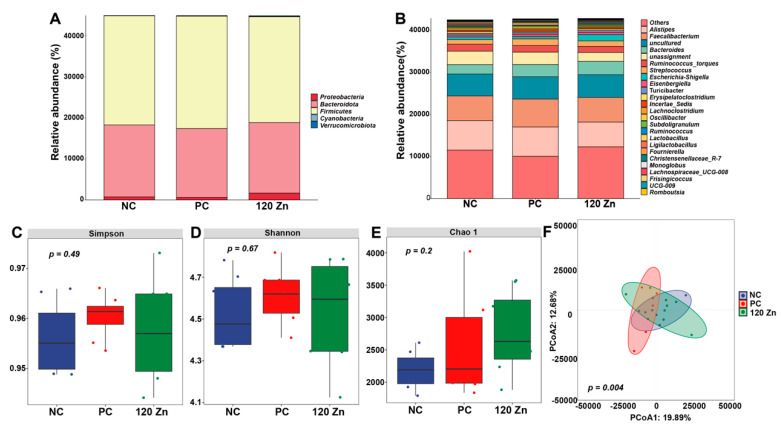
Relative abundance of the 5 phyla (**A**) and top 25 genera (**B**); alpha diversities, including Simpson index, Shannon index, and Chao 1 index (**C**–**E**); and principal coordinates analysis (PCoA) plots (**F**) in the three groups. n = 7–8. NC, negative control without treatment; PC, positive control with oral gavage of *C. perfringens*; 120 Zn, dietary supplemented with 120 mg/kg ZnO-QDs with oral gavage of *C. perfringens*.

**Figure 5:**
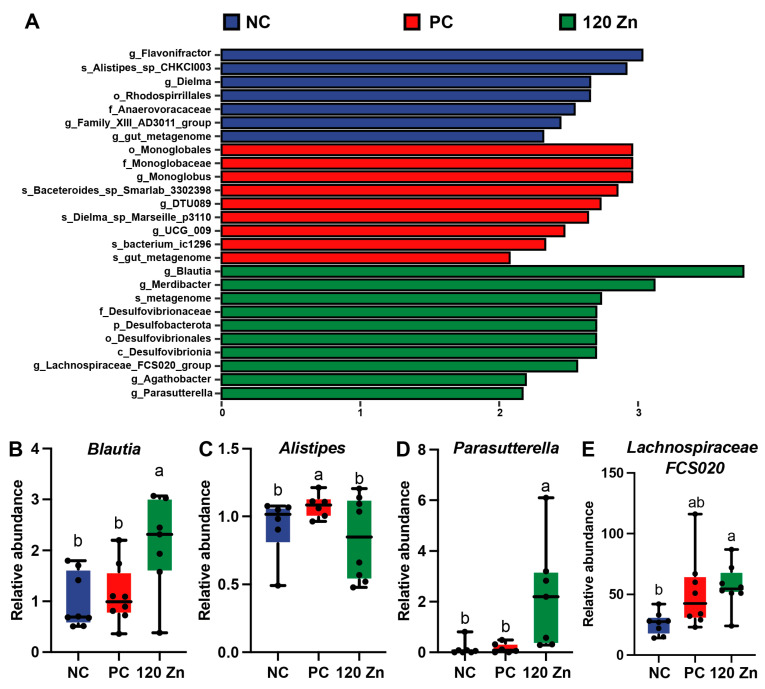
Differential bacterial features were identified by LEfSe analysis (**A**). Relative abundances of *Blautia*, *Alistipes*, *Parasutterella*, and *Lachnopiraceae FCS020* mong the three groups (**B**–**E**). Labeled means with different superscript letters are significantly different (*p* < 0.05) (n = 8). NC, negative control without treatment; PC, positive control with oral gavage of *C. perfringens*; 120 Zn, dietary supplemented with 120 mg/kg ZnO-QDs with oral gavage of *C. perfringens*.

A total of 5556 bacterial features, which were assigned to 10 known phyla and 238 genera, were identified by 16S rRNA sequencing (Supporting Information File). Of them, five phyla, *Proteobacteria*, *Bacteroidota*, *Firmicutes*, *Cyanobacteria*, and *Verrucomicrobiota*, were predominantly found (Figure 4A). The top eight most abundant microbial genera in the cecal microbiota of the broilers were *Alistipes*, *Faecalibacterium*, *Bacteroides*, *Ruminococcus*, *Streptococcus*, *Escherichia-Shigella*, *Eisenbergiella*, and *Turicibacter* (Figure 4B). Moreover, the Simpson, Shannon, and Chao1 indexes were not altered by *C. perfringens* infection and dietary ZnO-QD supplementation (Figure 4C–E). However, a significant separation (*p* = 0.004) was found in the cecal microbial profiles among the three groups through the PCoA analysis (Figure 4F). Further LEfSe analysis revealed that 11 significant differential genera were identified among the groups, such as *Blautia*, *Flavonifractor*, *Parasutterella*, *Dielma*, and *Monoglobus* (Figure 5A). Among these differential microorganisms, as expected, the 120 Zn group decreased (*p* < 0.05) the abundance of *Alistipes*, while it increased (*p* < 0.05) the abundance of *Blautia*, *Parasutterella*, and *Lachnospiraceae FCS020* when compared to the NC or PC groups (Figure 5B–E).

## 3. Discussion

Our previous study showed that ZnO-QDs in the diet can improve growth performance and promote the intestinal health of broilers [20]. Here, we investigated the effects of dietary ZnO-QDs on broiler chickens under *C. perfringens* challenge. The results indicate that the ZnO-QDs exhibited greater antibacterial activity compared to traditional ZnO. The MIC of the ZnO-QDs for *C. perfringens* was 0.413 mg/mL, and a 4 MIC dose eradicated *C. perfringens* within 10 h in vitro. On the other hand, dietary supplementation with ZnO-QDs could mitigate the adverse effects of growth performance induced by *C. perfringens* in broilers. Specifically, *C. perfringens* infection reduced the body weight of the broilers at 21 days. However, dietary supplementation with 40 and 120 mg Zn/kg ZnO-QDs alleviated the infection-induced reduction in body weight. Notably, no differences in serum LPS contents were found among the five groups, which might be due to the endotoxin originating from Gram-negative bacteria, whereas *C. perfringens* is Gram-positive [22,23]. Additionally, the DAO levels were increased in the infected group. The serum DAO level always serves as a biomarker indicative of intestinal barrier integrity [24]. Upon intestinal mucosal damage, DAO is released into systemic circulation due to compromised epithelial integrity. Dietary-supplemented 40–120 mg ZnO-QDs effectively alleviated intestinal lesions, and 40–80 mg Zn ZnO-QDs mitigated the increase in serum DAO levels induced by *C. perfringens*. These results indicate that ZnO-QDs effectively mitigate *C. perfringens* infection-induced negative effects in broilers, potentially through their antimicrobial properties and enhancement of gut health.

In general, the antioxidant system and the pro-oxidative system are always in balance, maintain a redox dynamic equilibrium, and scavenge excess free radicals in time to avoid oxidative damage. Previous research has demonstrated that intestinal oxidative stress, inflammation, and mucosal serious pathological changes occur in *C. perfringens*-challenged broilers [25,26]. In the present study, compared to the NC group, the PC group exhibited an imbalance in redox homeostasis, with significantly lower activities of CAT and SOD, and the MDA concentration slightly increased, indicating a state of oxidative stress in broilers under *C. perfringens* infection. In contrast, dietary-supplemented 40 mg Zn/kg ZnO-QDs enhanced the intestinal antioxidant activity by increasing the antioxidant enzymes CAT and GPX activities and decreasing MDA concentrations in the jejunum. These outcomes demonstrate that intestinal redox homeostasis was substantially disrupted in *C. perfringens*-challenged broilers, which could be alleviated by dietary supplementation with ZnO-QDs.

*C. perfringens* elicits host immune responses through the production of toxins and metabolic byproducts. Previous studies have highlighted that ZnO-QDs could improve intestinal immunity in broilers, thereby strengthening their antimicrobial defense capacity [19,20,27]. Antimicrobial peptides are essential for host defense and play a crucial role in limiting microbial infections [28]. Cathelicidins constitute a family of antimicrobial peptides with immunomodulatory and spectral antimicrobial activity [29]. Our results show that the *C. perfringens* reduced the *Cathelicidin-1* mRNA level, while 40–80 mg Zn/kg ZnO-QDs mitigates this change. Meanwhile, 120 mg Zn/kg ZnO-QD supplementation significantly reduced *Cathelicidin-1*, *Cathelicidin-2*, and *Cathelicidin-3* gene expression at the mRNA levels. This result is consistent with our previous findings, likely as ZnO-QDs share similar functions with antimicrobial peptides, causing the host’s adaptive mechanism to down-regulate them [20], which requires further exploration. Moreover, up-regulation of anti-inflammatory cytokines, such as *IL-10*, are often involved in the attenuation of the inflammatory response. Our results show dietary supplementation with ZnO-QDs significantly increased *IL-10* gene expression, suggesting that the preventive role of ZnO-QDs against *C. perfringens*-induced intestinal inflammation may be attributed to its capacity to up-regulate the expression of anti-inflammatory cytokines. Notably, IL-6 is a multifunctional cytokine that is involved in the immune response and the activation, growth, and differentiation of T cells that participate in the inflammatory response [30]. Elevated expression of *IL-6* can assist in identifying heterophil populations more capable of responding to and eliminating pathogens [31,32], which could explain why *IL-6* gene expression was up-regulated by ZnO-QDs treatment. Furthermore, our current study showed that *C. perfringens*-infected broilers reduced the expression of tight junction genes (*Claudin-1* and *Occludin*), but dietary supplementation of ZnO-QDs can alleviate the reduction of *Claudin-1*, as well as down-regulate intestinal *MLCK* gene expression. MLCK, an intracellular signal molecule, can promote intestinal actin and myosin filament contraction, open epithelial cell tight junction proteins, and subsequently regulate mucosal permeability of epithelial cells [33]. These outcomes suggest that the beneficial effects of ZnO-QDs against *C. perfringens* infection on the gut barrier function may be achieved partly by inhibiting MLCK gene expression.

The gut microbiota plays a crucial role in maintaining broiler intestinal health by promoting gut structural development, enhancing immunity, defending against pathogens, and facilitating nutrient digestion and utilization [34]. In this study, the superior intestinal health and reduced inflammatory status in the 120 Zn group compared to the PC group suggest that dietary 120 mg Zn/kg ZnO-QD supplementation may counteract *C. perfringens* infection by modulating specific microbial genera or species. Thus, 16S rRNA gene sequencing technology was utilized to analyze the cecal microbiota of broilers among the NC, PC, and 120 Zn groups. Strikingly, compared to the PC group, the 120 Zn group reduced the abundance of *Alistipes*, while it increased the abundance of *Blautia*, *Parasutterella*, and *Lachnospiraceae FCS020*. *Alistipes*, a relatively new bacterial genus belonging to the Bacteroidetes phylum, is highly relevant in chronic intestinal inflammation [35,36]. Thus, decreasing *Alistipes* by ZnO-QDs may improve the gut immunity status of broilers under *C. perfringens* infection. Moreover, *Blautia* is a dominant probiotic genus recognized for its dual functionality in biotransformation and interactions with other intestinal microbiota, and it also plays a significant role in maintaining intestinal environmental balance and mitigating inflammatory responses by up-regulating intestinal regulatory T cells [37,38]. *Parasutterella* exhibited an inverse correlation with inflammatory genes and a direct association with tight junction genes [39]. *Lachnospiraceae FCS020* is capable of producing short-chain fatty acids and is negatively correlated with the inflammatory cytokines and thiobarbituric acid reactive substances level [40]. Therefore, the increase in the three genera in the intestine by dietary ZnO-QD supplementation might contribute to the amelioration of intestinal inflammation and enhancement of epithelial barrier function. Further research into the specific roles of these genera and their interactions with ZnO-QDs will deepen our understanding of how gut microbiota contribute to broiler health under *C. perfringens* infection.

## 4. Conclusions

In summary, dietary supplementation of ZnO-QDs can moderately improve the growth performance of broilers under *C. perfringens* challenge. The protective mechanisms of ZnO-QDs against *C. perfringens* in broilers involve enhancing the redox status and tight junction in the jejunum, while simultaneously regulating the composition of gut microbiota. In conclusion, these results indicate that dietary ZnO-QD supplementation could be a viable nutritional strategy to protect broilers against *C. perfringens* in the future.

## 5. Materials and Methods

### 5.1. Experimental Materials

The ZnO-QDs were provided by the Sichuan Chelota Biotech Corporation Limited. The ZnO-QDs’ average size was approximately 4 nm, as described in our previous study [20]. *Escherichia coli K88*, *Salmonella pullorum* (ATCC13036), *Staphylococcus aureus* (CICC1001), and *C. perfringens* (CVCC2023) were obtained from the China Center of Industrial Culture Collection. The chicken *C. perfringens* type A field strain was obtained from the China Veterinary Culture Collection Center (Beijing, China) and cultured as previously described [41].

### 5.2. Antibacterial Activities In Vitro Experiment

ZnO-QDs and ZnO suspensions were prepared as previously described [42]. The broth microdilution method was used to determine the minimum inhibitory concentration (MIC) of ZnO-QDs and ZnO [43]. Briefly, serial twofold dilutions of ZnO-QDs (3.300 mg/mL to 0.006 mg/mL) and ZnO (7.600 mg/mL to 0.014 mg/mL) in Mueller Hinton (MH) broth were prepared in a 96-well plate with 100 μL per well. Each well received 80 μL of bacterial inoculum (1 × 10^6^ CFU/mL) and 20 μL of INT at a final concentration of 0.2 mg/mL, followed by incubation at 37 °C for 30 min. The MIC was read as the concentration of the ZnO-QDs and ZnO that completely inhibited visible bacterial growth.

For determining the bacteriostatic zone of ZnO-QDs against *C. perfringens*, 1 mL *C. perfringens* (1 × 10^6^ CFU/mL) were added into 100 mL of anaerobic meat liver broth and mixed. Seven wells (7 mm in diameter) were evenly placed on the surface of the solidified medium, then 15 mL of the bacterial suspensions were added. After the medium solidified, the evenly placed wells were taken out, and 100 μL ZnO-QD suspensions (1.650, 0.825, 0.413, 0.206, 0.026, 0.000 mg/mL) were added into the small holes, respectively. A 0.85% phosphoric acid was selected as the control group. After standing for 1 h, the samples were cultured in a 37 °C incubator for 24 h. The diameters of the bacteriostatic zones were measured using a vernier caliper. The growth curves of *E. coli*, *S. pullorum*, *S. aureus*, and *C. perfringens* treated with ZnO-QDs were measured as previously described [44], and the time-kill kinetics assay was performed according to a previous study [45].

### 5.3. Birds, Treatment, and Growth Performance

The animal trial was conducted following the approved protocol (HZAUCH-2024-0021). This study included 320 one-day-old Cobb male broilers with similar weights, which were randomly allocated into 5 groups, with 8 replicates of 8 broilers/cage. The broilers were fed a basal diet (Table S1) and had free access to water. The five experimental groups were as follows: negative control (NC), a basal diet without additives; positive control (PC), a basal diet with oral gavage of *C. perfringens*; and three treatment groups (40, 80, and 120 Zn), a basal diet supplemented with 40, 80, or 120 mg Zn/kg ZnO-QDs and oral gavage of *C. perfringens*. All the broilers (except the NC group) were orally gavaged with a broth culture of *C. perfringens* (1 mL per bird, 1 × 10^8^ CFU/mL) daily from 14 to 21 days, as previously described [41]. Each eight birds were housed in a separate cage (0.8 m × 0.8 m × 0.5 m) as one replicate. The feed intake and body weight of the broilers in each cage were weighted on days 0, 21, and 28 for the calculation of the average daily gain (ADG), average daily feed intake (ADFI), and feed conversion ratio (FCR). On the last day of the trial, eight birds from each group were randomly selected, weighed, and euthanized after 12 h of feed deprivation. Following this, the serum, jejunum, and cecal chyme were rapidly collected for biochemical or microbial analyses [46]. All the samples were stored at −80 °C until analysis.

### 5.4. Intestinal Lesion Score

The jejunum from each broiler was collected and evaluated for necrotic enteritis lesions using a scoring system ranging from zero to four, as previously described: 0, normal intestinal appearance; 1, thin-walled and friable intestines with small, red petechiae; 2, one to five dot-like lesions with diameters <1 mm; 3, sizable patches of necrosis, gas-filled intestine, and small flecks of blood; and 4, severe extensive necrosis, marked hemorrhage, and large amounts of gas in the intestine [47].

### 5.5. Serum Biochemistry and Jejunum Antioxidant Parameter Analysis

The contents of lipopolysaccharide (LPS) and diamine oxidase (DAO) in the serum were measured by using specific ELISA kits (MM-60017O2, MM-33277O2; Jiangsu Enzyme Immune Industrial, Nanjing, China) [48]. The jejunal activities of glutathione peroxidase (GPX), catalase (CAT), glutathione reductase (GR), malondialdehyde (MDA), and superoxide dismutase (SOD) were determined by commercialized reagent kits (A005-1, A007-1, A062-1, A003-1, A001-3; Jiancheng Bioengineering, Nanjing, China), as previously described [49].

### 5.6. Real-Time Quantitative PCR

Total RNA was extracted from the jejunum using TRIzol reagent (R401-01; Vazyme, Nanjing, China), as previously described [50]. The 2^−ΔΔCt^ method was applied for stage-related comparison of gene expression, and the relative abundance of the target genes was normalized to β-Actin [51]. The primers information is presented in Table S2.

### 5.7. Gut Microbiota Analysis

Cecal digesta samples from the NC, PC, and 120 Zn groups from this experiment were used for microbiome analysis. Briefly, the total bacterial DNA was isolated using the DNA stool mini kit (Tiangen, Beijing, China), and the V3–V4 region of the bacterial 16S rRNA gene was amplified with the primers 338F (50-ACTCCTACGGGAGGCAGCA-30) and 806R (50-GGACTACHVGGGTWVTAAT-30). The purified PCR products were sequenced on the Illumina Miseq platform. After sequencing, the raw sequences were filtered, denoised, and de-chimerized using the DADA2 plugin to avoid sequencing errors [52,53]. The microbial composition, indexes of diversity (Shannon, Simpson, and Chao 1), and principal coordinate analysis (PCoA), based on Jaccard distances, were calculated or generated using the OmicStudio tools V2.1 [54]. Further LDA effect size (LEfSe) analysis was applied to identify differential taxonomy among the groups [55].

### 5.8. Statistical Analysis

The results are presented as the mean ± SD. Data processing was conducted using SPSS Statistics 26. The data were tested by a normality test (Shapiro–Wilk test) to determine whether they were normally distributed. Differences between the two groups were assessed with the one-way analysis of variance (ANOVA), followed by Duncan’s multiple range test for multiple mean comparisons [56]. *p* < 0. 05 was considered statistically significant.

## Figures and Tables

**Table 1:** MIC ^1^ of ZnO-QDs and ZnO for four bacterial strains.

Strain	MIC (mg/mL)	
	ZnO-QDs	ZnO
*E. coli*	0.413	3.800
*S. Pulorum*	0.052	0.059
*S. aureus*	0.026	0.238
*C. perfringens*	0.413	1.900

^1^ MIC—Minimum inhibitory concentration.

**Table 2:** Growth performance of broilers infected by *C. perfringens* under dietary-supplemented ZnO-QDs ^1^.

	NC	PC	40 Zn	80 Zn	120 Zn
Day 0 BW, g/bird	45.05 ± 0.37	45.08 ± 0.52	45.12 ± 0.50	45.00 ± 0.31	44.86 ± 0.60
Day 21 BW, g/bird	740.91 ± 35.16 ^b^	717.18 ± 30.37 ^c^	757.95 ± 42.29 ^ab^	738.85 ± 30.79 ^bc^	775.70 ± 22.84 ^a^
Day 28 BW, g/bird	1256.07 ± 81.38	1210.32 ± 67.61	1205.90 ± 87.84	1262.47 ± 61.14	1252.01 ± 61.27
1 to 28 days					
ADG, g/d/bird	43.25 ± 1.03	41.62 ± 0.85	41.46 ± 1.11	43.48 ± 0.77	43.11 ± 0.78
ADFI, g/d/bird	65.78 ± 1.23	66.23 ± 1.08	63.26 ± 1.36	66.51 ± 1.31	65.2 ± 1.55
FCR, g/g	1.52 ± 0.07 ^b,#^	1.59 ± 0.08 ^a,#^	1.53 ± 0.09 ^ab^	1.53 ± 0.11 ^ab^	1.52 ± 0.12 ^ab^

^1^ Values are means ± SD, n = 8–10. Means in a row with different superscript lowercase letters are different, *p* < 0.05. ^#^ indicates a tendency, 0.05 ≤ *p* < 0.10. BW, body weight; ADFI, average daily feed intake; ADG, average daily gain; FCR, feed conversion ratio; NC, negative control without treatment; PC, positive control with oral gavage of *C. perfringens*; 40 Zn, 80 Zn, and 120 Zn, dietary supplementation of 40, 80, and 120 mg/kg ZnO-QDs with oral gavage of *C. perfringens*.

**Table 3:** Effect of ZnO-QDs on the antioxidant status of broilers infected by *C. perfringens* ^1^.

	NC	PC	40 Zn	80 Zn	120 Zn
CAT, U/mg protein	0.76 ± 0.10 ^a^	0.57 ± 0.18 ^bc^	0.75 ± 0.18 ^ab^	0.45 ± 0.15 ^c^	0.61 ± 0.18 ^bc^
SOD, U/mg protein	78.86 ± 22.82 ^a^	46.75 ± 10.04 ^b^	60.24 ± 20.04 ^ab^	48.13 ± 8.26 ^b^	62.73 ± 26.89 ^ab^
GPX, U/mg protein	1.50 ± 0.58 ^b^	1.25 ± 0.69 ^b^	2.60 ± 1.18 ^a^	1.80 ± 0.78 ^ab^	2.42 ± 1.10 ^a^
GR, U/g protein	5.71 ± 2.68	3.64 ± 1.69	4.34 ± 2.06	5.92 ± 3.17	4.47 ± 1.75
MDA, nmol/mg protein	2.47 ± 0.94 ^ab^	3.07 ± 0.99 ^a^	2.15 ± 0.6 ^b^	1.79 ± 0.41 ^b^	2.27 ± 1.02 ^ab^

^1^ Values are means ± SD, n = 8–10. Different letters between groups represent significant differences, *p* < 0.05. CAT, catalase; SOD, superoxide dismutase; GPX, glutathione peroxidase; GR, glutathione reductase; MDA, malondialdehyde; NC, negative control without treatment; PC, positive control with oral gavage of *C. perfringens*; 40 Zn, 80 Zn, and 120 Zn, dietary supplementation of 40, 80, and 120 mg/kg ZnO-QDs with oral gavage of *C. perfringens*.

## Data Availability

The original contributions presented in this study are included in the article/Supplementary Material. Further inquiries can be directed to the corresponding authors.

## Supplementary Materials

The following supporting information can be downloaded at: https://www.mdpi.com/article/10.3390/toxins17060272/s1, Table S1: Ingredients and nutrient composition of the basal diet; Table S2: Sequences for real-time PCR primers.

## References

[1] Lee K.W., Lillehoj H.S. (2021). Role of Clostridium perfringens Necrotic Enteritis B-like Toxin in Disease Pathogenesis. Vaccines.

[2] Caly D.L., D’Inca R., Auclair E., Drider D. (2015). Alternatives to Antibiotics to Prevent Necrotic Enteritis in Broiler Chickens: A Microbiologist’s Perspective. Front. Microbiol..

[3] Broom L.J. (2017). Necrotic enteritis; current knowledge and diet-related mitigation. World Poult. Sci. J..

[4] Emami N.K., Calik A., White M.B., Young M., Dalloul R.A. (2019). Necrotic Enteritis in Broiler Chickens: The Role of Tight Junctions and Mucosal Immune Responses in Alleviating the Effect of the Disease. Microorganisms.

[5] Li J., Adams V., Bannam T.L., Miyamoto K., Garcia J.P., Uzal F.A., Rood J.I., McClane B.A. (2013). Toxin plasmids of Clostridium perfringens. Microbiol. Mol. Biol. Rev..

[6] Kiu R., Hall L.J. (2018). An update on the human and animal enteric pathogen Clostridium perfringens. Emerg Microbes Infect..

[7] Moore R.J. (2024). Necrotic enteritis and antibiotic-free production of broiler chickens: Challenges in testing and using alternative products. Anim. Nutr..

[8] Hofacre C.L., Smith J.A., Mathis G.F. (2018). An optimist’s view on limiting necrotic enteritis and maintaining broiler gut health and performance in today’s marketing, food safety, and regulatory climate. Poult. Sci..

[9] Cao K.X., Deng Z.C., Li S.J., Yi D., He X., Yang X.J., Guo Y.M., Sun L.H. (2024). Poultry Nutrition: Achievement, Challenge, and Strategy. J. Nutr..

[10] Zhu C., Lv H., Chen Z., Wang L., Wu X., Chen Z., Zhang W., Liang R., Jiang Z. (2017). Dietary Zinc Oxide Modulates Antioxidant Capacity, Small Intestine Development, and Jejunal Gene Expression in Weaned Piglets. Biol. Trace Elem. Res..

[11] Grilli E., Tugnoli B., Vitari F., Domeneghini C., Morlacchini M., Piva A., Prandini A. (2015). Low doses of microencapsulated zinc oxide improve performance and modulate the ileum architecture, inflammatory cytokines and tight junctions expression of weaned pigs. Animal.

[12] Radi A.M., Abdel Azeem N.M., El-Nahass E.S. (2021). Comparative effects of zinc oxide and zinc oxide nanoparticle as feed additives on growth, feed choice test, tissue residues, and histopathological changes in broiler chickens. Environ. Sci. Pollut. Res. Int..

[13] Ramiah S.K., Awad E.A., Mookiah S., Idrus Z. (2019). Effects of zinc oxide nanoparticles on growth performance and concentrations of malondialdehyde, zinc in tissues, and corticosterone in broiler chickens under heat stress conditions. Poult. Sci..

[14] Raghupathi K.R., Koodali R.T., Manna A.C. (2011). Size-dependent bacterial growth inhibition and mechanism of antibacterial activity of zinc oxide nanoparticles. Langmuir.

[15] Jones N., Ray B., Ranjit K.T., Manna A.C. (2008). Antibacterial activity of ZnO nanoparticle suspensions on a broad spectrum of microorganisms. FEMS Microbiol. Lett..

[16] Nair S., Sasidharan A., Divya Rani V.V., Menon D., Nair S., Manzoor K., Raina S. (2009). Role of size scale of ZnO nanoparticles and microparticles on toxicity toward bacteria and osteoblast cancer cells. J. Mater. Sci. Mater. Med..

[17] Happy A., Soumya M., Venkat Kumar S., Rajeshkumar S. (2018). Mechanistic study on antibacterial action of zinc oxide nanoparticles synthesized using green route. Chem. Biol. Interact..

[18] Kumar Verma A. (2022). ZnO quantum dots a novel nanomaterial for various applications: Recent advances and challenges. Indian J. Biochem. Biophys..

[19] Li Y., Xie S., Xu D., Shu G., Wang X. (2021). Antibacterial activity of ZnO quantum dots and its protective effects of chicks infected withSalmonella pullorum. Nanotechnology.

[20] Shi L., Ruan M.L., Zhang B.B., Gong G.X., Li X.W., Refaie A., Sun L.-H., Deng Z.-C. (2024). Effects of Dietary Supplementation of Zinc Oxide Quantum Dots on Growth Performance and Gut Health in Broilers. Biol. Trace Elem. Res..

[21] Du F., Niu J., Hong Y., Fang X., Geng Z., Liu J., Xu F., Liu T., Chen Q., Zhai J. (2025). Microwave-Assisted Synthesized ZnO@APTES Quantum Dots Exhibits Potent Antibacterial Efficacy Against Methicillin-Resistant Staphylococcus aureus without Inducing Resistance. Int. J. Nanomed..

[22] Maldonado R.F., Sa-Correia I., Valvano M.A. (2016). Lipopolysaccharide modification in Gram-negative bacteria during chronic infection. FEMS Microbiol. Rev..

[23] Mak P.H.W., Rehman M.A., Kiarie E.G., Topp E., Diarra M.S. (2022). Production systems and important antimicrobial resistant-pathogenic bacteria in poultry: A review. J. Anim. Sci. Biotechnol..

[24] Zhang L., Fan X., Zhong Z., Xu G., Shen J. (2015). Association of plasma diamine oxidase and intestinal fatty acid-binding protein with severity of disease in patient with heat stroke. Am. J. Emerg. Med..

[25] Zhang X., Zhao Q., Ci X., Chen S., Xie Z., Li H., Zhang H., Chen F., Xie Q. (2020). Evaluation of the efficacy of chlorogenic acid in reducing small intestine injury, oxidative stress, and inflammation in chickens challenged with Clostridium perfringens type A. Poult. Sci..

[26] Liu N., Lin L., Wang J., Zhang F., Wang J.P. (2018). Dietary cysteamine hydrochloride protects against oxidation, inflammation, and mucosal barrier disruption of broiler chickens challenged with Clostridium perfringens. J. Anim. Sci..

[27] Zhou Z., Zhang T., Chen Y., Zhou X., Zhong Y., Liu H., Zhong Z., Hu Y., Liao F., Wang X. (2023). Zinc Oxide Quantum Dots May Provide a Novel Potential Treatment for Antibiotic-Resistant Streptococcus agalactiae in Lama glama. Molecules.

[28] Mookherjee N., Anderson M.A., Haagsman H.P., Davidson D.J. (2020). Antimicrobial host defence peptides: Functions and clinical potential. Nat. Rev. Drug Discov..

[29] Kosciuczuk E.M., Lisowski P., Jarczak J., Strzalkowska N., Jozwik A., Horbanczuk J., Krzyżewski J., Zwierzchowski L., Bagnicka E. (2012). Cathelicidins: Family of antimicrobial peptides. A review. Mol. Biol. Rep..

[30] Waititu S.M., Yitbarek A., Matini E., Echeverry H., Kiarie E., Rodriguez-Lecompte J.C., Nyachoti C.M. (2014). Effect of supplementing direct-fed microbials on broiler performance, nutrient digestibilities, and immune responses. Poult. Sci..

[31] Swaggerty C.L., Kogut M.H., Ferro P.J., Rothwell L., Pevzner I.Y., Kaiser P. (2004). Differential cytokine mRNA expression in heterophils isolated from Salmonella-resistant and -susceptible chickens. Immunology.

[32] Gadde U., Oh S.T., Lee Y.S., Davis E., Zimmerman N., Rehberger T., Lillehoj H.S. (2017). The Effects of Direct-fed Microbial Supplementation, as an Alternative to Antibiotics, on Growth Performance, Intestinal Immune Status, and Epithelial Barrier Gene Expression in Broiler Chickens. Probiotics Antimicrob. Proteins.

[33] Fazal F., Gu L., Ihnatovych I., Han Y., Hu W., Antic N., Carreira F., Blomquist J.F., Hope T.J., Ucker D.S. (2005). Inhibiting myosin light chain kinase induces apoptosis in vitro and in vivo. Mol. Cell. Biol..

[34] Yadav S., Jha R. (2019). Strategies to modulate the intestinal microbiota and their effects on nutrient utilization, performance, and health of poultry. J. Ani. Sci. Biotech..

[35] Moschen A.R., Gerner R.R., Wang J., Klepsch V., Adolph T.E., Reider S.J., Hackl H., Pfister A., Schilling J., Moser P.L. (2016). Lipocalin 2 Protects from Inflammation and Tumorigenesis Associated with Gut Microbiota Alterations. Cell Host Microbe.

[36] Parker B.J., Wearsch P.A., Veloo A.C.M., Rodriguez-Palacios A. (2020). The Genus Alistipes: Gut Bacteria with Emerging Implications to Inflammation, Cancer, and Mental Health. Front. Immunol..

[37] Liu X., Mao B., Gu J., Wu J., Cui S., Wang G., Zhao J., Zhang H., Chen W. (2021). Blautia-a new functional genus with potential probiotic properties?. Gut Microbes.

[38] Kim C.H., Park J., Kim M. (2014). Gut microbiota-derived short-chain Fatty acids, T cells, and inflammation. Immune Netw..

[39] Yang D.F., Huang W.C., Wu C.W., Huang C.Y., Yang Y.C.S.H., Tung Y.T. (2023). Acute sleep deprivation exacerbates systemic inflammation and psychiatry disorders through gut microbiota dysbiosis and disruption of circadian rhythms. Microbiol. Res..

[40] Hu R., Wu S., Li B., Tan J., Yan J., Wang Y., Tang Z., Liu M., Fu C., Zhang H. (2022). Dietary ferulic acid and vanillic acid on inflammation, gut barrier function and growth performance in lipopolysaccharide-challenged piglets. Anim. Nutr..

[41] Du E., Gan L., Li Z., Wang W., Liu D., Guo Y. (2015). In vitro antibacterial activity of thymol and carvacrol and their effects on broiler chickens challenged with Clostridium perfringens. J. Anim. Sci. Biotechnol..

[42] Orou S.F.C., Hang K.J., Thien M.T., Ying Y.L., Diem N.D.N., Goh B.H., Pung S.Y., Pung Y.F. (2018). Antibacterial activity by ZnO nanorods and ZnO nanodisks: A model used to illustrate “Nanotoxicity Threshold”. J. Ind. Eng. Chem..

[43] Zhang S., Wang P., Shi X., Tan H. (2021). Inhibitory properties of Chinese Herbal Formula SanHuang decoction on biofilm formation by antibiotic-resistant Staphylococcal strains. Sci. Rep..

[44] Wu Y., Bai J., Zhong K., Huang Y., Gao H. (2017). A dual antibacterial mechanism involved in membrane disruption and DNA binding of 2R,3R-dihydromyricetin from pine needles of Cedrus deodara against Staphylococcus aureus. Food Chem..

[45] Ren G., Hu D., Cheng E.W., Vargas-Reus M.A., Reip P., Allaker R.P. (2009). Characterisation of copper oxide nanoparticles for antimicrobial applications. Int. J. Antimicrob. Agents.

[46] Deng Z.C., Wang J., Wang J., Yan Y.Q., Huang Y.X., Chen C.Q., Sun L., Liu M. (2024). Tannic acid extracted from gallnut improves intestinal health with regulation of redox homeostasis and gut microbiota of weaned piglets. Anim. Res. One Health.

[47] Liu D., Guo S., Guo Y. (2012). Xylanase supplementation to a wheat-based diet alleviated the intestinal mucosal barrier impairment of broiler chickens challenged by Clostridium perfringens. Avian Pathol..

[48] Wang S.Q., Peng Z., Sun H., Han Y.M., Zhang B., Pineda L., Boerboom G., Sun L.-H., Liu Y., Deng Z.-C. (2024). Evaluating the Impact of an Organic Trace Mineral mix on the Redox Homeostasis, Immunity, and Performance of Sows and their Offspring. Biol. Trace Elem. Res..

[49] Liu M., Li X.W., Sun H., Yan Y.Q., Xia Z.Y., Refaie A., Zhang N.-Y., Wang S., Tan C., Sun L.-H. (2025). T-2 toxin-induced splenic injury by disrupting the gut microbiota-spleen axis via promoting IL-6/JAK/STAT1 signaling-mediated inflammation and apoptosis and its mitigation by elemental nano-selenium. Arch. Toxicol..

[50] Yang J.C., Liu M., Huang R.H., Zhao L., Niu Q.J., Xu Z.J., Wei J.-T., Lei X.G., Sun L.-H. (2024). Loss of SELENOW aggravates muscle loss with regulation of protein synthesis and the ubiquitin-proteasome system. Sci. Adv..

[51] Yan Y.Q., Liu M., Xu Z.J., Xu Z.J., Huang Y.X., Li X.M., Chen C.-J., Zuo G., Yang J.-C., Lei X.G. (2024). Optimum Doses and Forms of Selenium Maintaining Reproductive Health via Regulating Homeostasis of Gut Microbiota and Testicular Redox, Inflammation, Cell Proliferation, and Apoptosis in Roosters. J. Nutr..

[52] Chen S., Zhou Y., Chen Y., Gu J. (2018). fastp: An ultra-fast all-in-one FASTQ preprocessor. Bioinformatics.

[53] Rognes T., Flouri T., Nichols B., Quince C., Mahe F. (2016). VSEARCH: A versatile open source tool for metagenomics. PeerJ.

[54] Lyu F., Han F., Ge C., Mao W., Chen L., Hu H., Chen G., Lang Q., Fang C. (2023). OmicStudio: A composable bioinformatics cloud platform with real-time feedback that can generate high-quality graphs for publication. Imeta.

[55] Cao K.X., Deng Z.C., Liu M., Huang Y.X., Yang J.C., Sun L.H. (2023). Heat Stress Impairs Male Reproductive System with Potential Disruption of Retinol Metabolism and Microbial Balance in the Testis of Mice. J. Nutr..

[56] Deng J., Peng Z., Xia Z., Mo Y., Guo L., Wei J., Sun L., Liu M. (2025). Five glutathione S-transferase isozymes played crucial role in the detoxification of aflatoxin B1 in chicken liver. J. Anim. Sci. Biotechnol..

