# Supplementary material for: Dietary Supplementation of Zinc Oxide Quantum Dots Protective Against Clostridium perfringens Induced Negative Effects in Broilers
Source: Toxins (Basel). 2025 May 29;17(6):272. doi: 10.3390/toxins17060272 (PMC12197405; doi:10.3390/toxins17060272)
Supplement: Supplementary file 1 [file toxins-17-00272-s001.zip › Supplemental Tables.pdf]

Table S1 Ingredients and nutrient composition of the basal diet

Ingredients	Days 1–21	Days 22–28	Nutrient level	Days 1–21	Days 22–28
Corn, %	60.5	63.1	ME, kcal/kg	2974.00	3079.00
Soybean meal, %	29.5	26	Crude protein, %	21.40	19.90
Wheat bran, %	3	3	Lysine, %	1.35	1.37
Soybean oil, %	2	3.13	Methionine, %	0.55	0.52
Dicalcium phosphate, %	2.1	2	Methionine + cystine, %	0.90	0.88
Calcium carbonate, %	1.3	1.1	Calcium, %	1.03	0.92
Choline chloride, %	0.2	0.3	Total phosphorus, %	0.69	0.66
Salt, %	0.3	0.3	Nonphytate P, %	0.54	0.51
Lysine, %	0.37	0.39			
Methionine, %	0.2	0.18			
Threonine, %	0.23	0.2			
Vitamin premix[1] , %	0.2	0.2			
Mineral premix[2] , %	0.1	0.1			
Total, %	100	100			

[1] Supplied vitamin per kg of the feed: vitamin A, 2000 IU; vitamin D3, 300 IU; vitamin E, 20 IU; vitamin B12, 0.02 mg; riboflavin, 1.4 mg; pantothenic acid, 2 mg; nicotinic acid, 7 mg; pyridoxine, 0.25 mg; folic acid, 0.15 mg; menadione, 0.3 mg; thiamin, 0.15 mg.

[2] Supplied minerals per kg of the feed: Cu, 8 mg; Mn, 60 mg; Fe, 80 mg; I, 0.35 mg; Se, 0.34 mg. Mineral premix was free of zinc.

[3] Nutrient levels were a calculated value.

Table S2 Sequences for real-time PCR primers

Gene	Accession No.	Primer sequences (5'-3')
Cathelicidin-1	NM_001001605.3	F: TGGCCGCTGGTCATCAG
		R: TTCTTGATCGCCCGGTAGAG
Cathelicidin-2	NM_001024830.2	F: CCGGGCGTCGATCTGA
		R: GGTGCACTCTGTCTCCATGATG
Cathelicidin-3	NM_001311177.1	F: CGATGTCACCTGCGTGGAC
		R: TTCTCCTGATGGCTTTGTAGAGGT
IL-2	AF000631.1	F: AGTGCACCCAGCAAACTCTG
		R: TCCGGTGTGATTTAGACCCGT
IL-6	NM_204628.2	F: TTCACCGTGTGCGAGAACAGC
		R: CAGCCGTCCTCCTCCGTCAC
IL-10	NM_001004414.4	F: CCAGCGAGTTCACCCTGAAT
		R: CGTCTGGTGTTTGCAGTTGG
Claudin-1	NM_001013611	F: GGTATGGCAACAGAGTGGCT
		R: CAGCCAATGAAGAGGGCTGA
Claudin-2	NM_001277622.1	F: CCTACATTGGTTCAAGCATCGTGA
		R: GATGTCGGGAGGCAGGTTGA
Occludin	NM_205128.1	F: AGTTCGACACCGACCTGAAG
		R: TCCTGGTATTGAGGGCTGTC
ZO-1	XM_015278975.4	F: TATGAAGATCGTGCGCCTCC
		R: GAGGTCTGCCATCGTAGCTC
MLCK	NM_001322361.3	F: GTTCCCCGCAGCTCTGTC
		R: CATCCCCCATGATGTGGACC
β-actin	NM_205518.1	F: GTCCACCGCAAATGCTTCTAA
		R: TGCGCATTTATGGGTTTTGTT
